# Grape expectations: disentangling environmental drivers of microbiome establishment in winegrowing ecosystems

**DOI:** 10.1038/s41522-026-00915-x

**Published:** 2026-01-16

**Authors:** Lena Flörl, Patrik Schönenberger, Markus Rienth, Nicholas A. Bokulich

**Affiliations:** 1 Department of Health Sciences and Technology, ETH Zurich, Zurich, Switzerland; 2 Changins College for Viticulture and Enology, Changins—University of Sciences and Art Western Switzerland, Nyon, Switzerland

**Keywords:** Environmental microbiology, Food microbiology, Microbial ecology, Applied microbiology

## Abstract

Microbial communities play a central role in viticulture, influencing wine characteristics (a concept termed *microbial terroir*). Yet, the individual factors shaping these microbiomes remain poorly understood. We conducted a multi-year, large-scale survey of Swiss vineyards (95 sites, 680 samples), longitudinally sampling 12 sites (within 2.46 km and identical cultivar and rootstock) over three years. Using 16S rRNA gene and internal transcribed spacer (ITS) amplicon sequencing, untargeted metabolomics (GC-MS, LC-MS/MS), environmental monitoring, and sensory data, we disentangled environmental factors associated with community assembly and fermentation dynamics. Topography and climate collectively structured microbiomes but affected soil- and plant-associated communities differently. Berry-associated fungi showed the strongest site-specific signature, enabling machine-learning predictions of microclimatic variation. Climatic factors and berry chemistry selectively favor fermentative yeasts, which are each linked to distinct metabolite and aroma profiles. Plant stress metabolites were further associated with microbial and metabolite composition. Our integrative approach thereby fundamentally advances our understanding of microbial biogeography and *terroir* in viticulture.

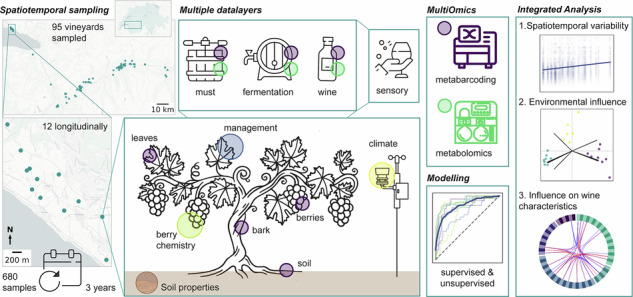

## Introduction

Microorganisms and microbial communities interact with plants as pathogens or beneficial partners, enhancing growth, nutrient uptake, stress tolerance, and disease resistance. Plant-associated microbiomes are shaped by the host genotype as well as a plethora of environmental factors, which are notoriously difficult to disentangle^1^. Grapevines (*Vitis vinifera*) are a unique biogeographic model for investigating associated microbial community dynamics. As long-lived perennials cultivated across the globe in diverse environments, grapevines undergo annual recolonization of plant organs, providing an ideal system to study microbial dispersal, niche adaptation, and temporal stability. Additionally, grape berry-associated microbes can directly impact fermentation and influence resulting wine characteristics, which makes vineyard and wine microbiomes an interesting system for studying microbial dynamics and transmission in fermented foods^2^. These contributions of regionally variable microbiota to wine characteristics is termed *microbial terroir*^3^, and extends our traditional understanding of *terroir* – encompassing the influence of site-specific factors including environmental variables (e.g. soil properties, climate), cultivar, rootstock, and management practices. Vineyard microbiomes are directly influenced by multiple biotic and abiotic factors, including climate and vintage effects^4^, cultivar^5–7^ and rootstock^8^, management practices^9,10^, altitude^11^, soil chemistry^12^, and surrounding land use^13^; and are predictive of wine quality downstream^4,14^. Yet, while numerous studies from major wine-growing regions worldwide have identified microbial patterns in the context of individual environmental factors and for different plant organs, a comprehensive understanding of specific contributions and underlying casualties of site-specific factors on vineyard microbial communities as a whole remains elusive.

In this study, we aimed to disentangle spatial, climatic, and biotic factors influencing microbial assembly in vineyards, and their impact on resulting wine properties. We hypothesize that (i) the microbial communities of different ecological niches in vineyards (soil, bark, leaves, berries) exhibit distinct spatiotemporal variation, (ii) this variation is shaped by a dynamic interaction of abiotic factors and (iii) that these variable berry-associated microbiota impact the resulting wine characteristics. Therefore, we conducted the first large-scale, systematic survey of regionally varying microbiota in Swiss vineyards, sampling a total of 95 vineyards. From these, we collected samples longitudinally from 12 vineyards in close geographic proximity, within a maximum distance of ~2.5 km from each other, over three consecutive years. By integrating detailed environmental data from this highly controlled setting with identical cultivars and rootstocks, under different microclimatic conditions and topographical features (altitude, solar exposure, etc.), while accounting for for several covariates that can be challenging to control in commercial vineyards. We collected samples from the same grape variety (Chasselas) grafted on identical rootstocks across distinct locations, and each year performed microvinifications. Samples were analyzed with three distinct untargeted metabolomics methodologies and sensory analysis. By analyzing these diverse data layers and multi-omics datasets using machine learning and integrated analysis, we gained new insights into microbial community assembly as a function of site, revealing dominant environmental influences and downstream effects on wine characteristics. This work thereby enhances our understanding of microbial contributions to *terroir* and provides fundamental insights into plant-microbiome interactions under variable environmental conditions.

## Results

We aimed to identify the environmental factors shaping microbial community assembly in vineyards, while controlling for biological variables that often confound microbial biogeography research in commercial agriculture. Here, we focus on an existing vineyard network in the Appellation d’origine contrôlée (AOC) Lavaux that has been deeply characterized for phenology, climate, pedology, and *terroir* expression^15^, planted exclusively to Chasselas cultivar and 3309C rootstock to control for biotic factors influencing microbial community assembly in grapevines^5,7^. Integrating microbiome analysis, metabolome analysis, and continuous environmental monitoring in this vineyard network thus provides an exquisite opportunity to study how spatial, environmental, climatic, and microbial factors interact with each other and with wine quality parameters downstream.

### Pedoclimatic gradient across studied vineyards

**Fig. 1:**
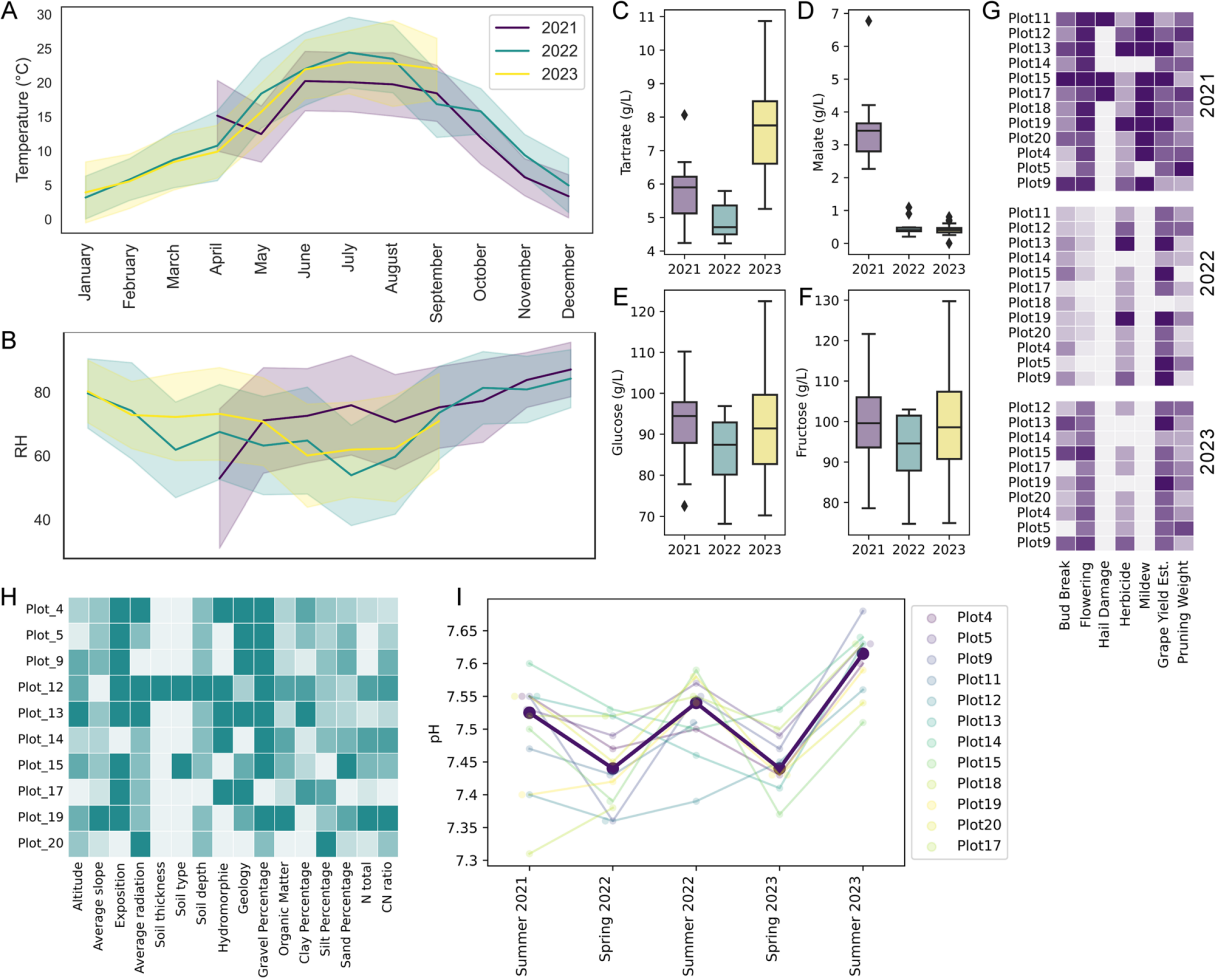
Spatiotemporal variability in climate, soil properties, and grape chemistry across AOC Lavaux vineyards. Characterization of the 12 vineyards sampled in AOC Lavaux over three years, illustrating climatic variation (**A**: temperature, **B**: relative humidity (RH)) and berry chemistry (**C**: tartrate, **D**: malate, **E**: glucose, **F**: fructose). Additionally, key agronomic and phenological metrics were assessed annually (**G**), including bud break and flowering time, hail damage, mildew occurrence, grape yield estimates, and pruning weight as a proxy for plant vigor. **H** All vineyards exhibited distinct pedoclimatic properties, including altitude, slope, solar exposure, radiation, soil type and depth, hydromorphy, geology, gravel percentage, organic matter and nitrogen content, and soil texture. **I** Soil pH was also measured throughout the years, revealing seasonal fluctuations. For the heatmaps (**G**, **H**) numerical values were normalized within the variable and categorical variables encoded: Hail Damage: : yes = 1, no = 0, Mildew: yes = 1, no = 0; Grape Yield Estimate: high = 3, medium = 2, low = 1, missing = 0; Herbicide: total = 3, almost total = 2, band = 1, missing = 0; Exposition: South = 0, South-West = 1; Soil type: Calcaire = 0, Calcique = 1; Soil thickness: calcareous = 0, colluvial = 1; Soil depth: 40–100 = 0, 100–180 = 1, above 150 = 2; Hydromorphie: Slightly redox = 1, no redox = 0; Geology: Bottom moraine = 0, Colluvium = 1, ravel moraine = 2, Molasse = 3; Gravel Percentage: 10–30% = 1, < 10% = 0, > 30% = 2.

First, we collected samples and data over three years from 12 distinct vineyards planted with Chasselas in the AOC Lavaux subregion “Villette”, situated within a radius of 2.46 km. The plots were chosen to represent distinct pedoclimatic characteristics present within the AOC, which are known to contribute to the specific environmental context of a vineyard such as inclination, altitude, and solar radiation. Additionally, we continuously measured temperature and relative humidity at each site and assessed soil characteristics, phenology, management practices and other agronomic information (e.g., spread of mildew, or hail damage). From the climate measurements, we calculated the daily median values and coefficient of variation to assess fluctuations per vineyard over the growing season. This revealed that 2021 was substantially more humid and cool (median relative humidity (RH): 71.98%, median temperature: 18.51 °C) than 2022 and 2023 (median RH: 63.39% and 65.71%, temperature: 20.81 °C and 20.58 °C, respectively). This variability is further reflected in the berry chemistry composition, e.g., substantially higher malate concentrations in 2021 due to cooler temperatures and consequently reduced malate respiration during ripening. In contrast, the average berry composition in 2022 and 2023 remained relatively consistent. This reflects the large impact of annual variations on berry development and chemical composition, which depend on the respective site-specific and climatic conditions (see Fig. 1).

### Spatio-temporal patterns of vineyard microbiomes

In total, we collected 680 samples, consisting of 441 berry samples, 98 samples from subsequent microvinifications (laboratory-scale fermentations conducted from these berries, see Methods), 55 soil samples, 53 leaf samples and 33 bark samples (see Supplementary Table 1). Samples were analyzed using marker-gene amplicon sequencing for bacterial (16S rRNA gene) and fungal (ITS) communities, yielding 18,795,287 reads for fungi and 3,306,142 reads for bacteria. Due to low sequencing depth for bacterial communities in most grape and leaf samples (mean read count <700), these samples were excluded from the beta diversity analysis. These sample types represent distinct ecological niches in vineyards, and exhibit significant differences in microbial alpha diversity (Kruskal Wallis for Fungi: p_Richness_ = 2.30e-39, *p*_Eveness_ = 1.56e-46, *p*_Shannon Entropy_ = 1.02e-42; Bacteria: *p*_Richness_ = 8.48e-19, *p*_Eveness_ = 2.15e-10, *p*_Shannon Entropy_ = 2.73e-17) with a gradient in richness from soil to bark to leaves to berries; and beta diversity, with soil being most distinct from annually newly formed plant organs like leaves and berries. The latter also showed substantial overlap, particularly in the presence of fungi (see Supplementary Fig. 1).

Next, we analyzed spatio-temporal variation in fungal and bacterial alpha and beta diversity of individual sample types within and between vineyard sites. In soils, significant spatiotemporal variation was observed in bacterial community evenness and abundance (Supplementary Table 2), but not for fungi (Supplementary Table 3). Yet, the composition of both fungal and bacterial communities exhibited significant variation across different vineyards and sampling time points (spring, summer), as determined by Permutational Analysis of Variance (PERMANOVA). (Table 1, Table 2). Notably, the phylogenetic structure of fungal communities significantly varied between timepoints and years, even when comparing samples from the same vineyard soils. The vineyard had the overall strongest influence on soil community composition, accounting for 27.3% to 38.7% of the variation in bacterial communities and 30.5% to 39.8% in fungal communities, with abundance-weighted metrics leading to a higher proportion of the explained variation attributed to site effects. However, no significant distance-decay relationship was observed using Mantel tests (Supplementary Table 4), indicating that spatial proximity was not associated with microbial community similarity in these soils. Similarly to soil, fungal communities of bark significantly varied between vineyards, with up to 45% of variance explained by site, but without physical proximity of vineyards significantly contributing to this variation (Supplementary Table 5). These communities appear to be temporally stable as no significant differences were detected between years (Table 1). In contrast, bacterial communities did not significantly vary by location, but their phylogenetic composition differed substantially between years.

**Table 1:** Permutational analysis of variance (PERMANOVA) of the various beta diversity metrics (Bray-Curtis, Jaccard, Bray-Curtis of k-mers, Jaccard of k-mers) of fungal communities for the different vineyard sample types (berries, soil, bark and leaves)

	Bray Curtis		Jaccard		k-mer: Bray Curtis		k-mer: Jaccard	
	R2	p-value*	R2	p-value*	R2	p-value*	R2	p-value*
**Berries**								
Plot ID	0.106	**0.001**	0.051	**0.001**	0.161	**0.001**	0.078	**0.001**
Year	0.015	**0.001**	0.012	**0.001**	0.027	**0.001**	0.025	**0.001**
Time point	0.028	**0.001**	0.006	**0.001**	0.034	**0.001**	0.013	**0.001**
Plot ID : Year	0.043	**0.003**	0.033	**0.001**	0.057	**0.001**	0.038	**0.001**
Plot ID : Time point	0.044	**0.006**	0.026	0.993	0.051	**0.001**	0.032	**0.004**
Year : Time point	0.018	**0.001**	0.003	**0.044**	0.022	**0.001**	0.004	**0.022**
Residuals	0.746	NaN	0.869	NaN	0.647	NaN	0.811	NaN
**Soil**								
Plot ID	0.340	**0.001**	0.305	**0.001**	0.398	**0.001**	0.375	**0.001**
Year	0.028	**0.002**	0.027	**0.001**	0.031	**0.001**	0.031	**0.001**
Time point	0.047	**0.001**	0.028	**0.001**	0.064	**0.001**	0.035	**0.001**
Plot ID : Year	0.214	0.245	0.239	0.105	0.195	0.043	0.216	**0.047**
Plot ID : Time point	0.205	0.064	0.213	0.286	0.180	**0.033**	0.182	0.368
Year : Time point	0.017	0.702	0.021	0.437	0.016	0.262	0.018	0.421
Residuals	0.148	NaN	0.167	NaN	0.116	NaN	0.142	NaN
**Bark**^a^								
Plot ID	0.433	**0.049**	0.400	**0.015**	0.450	**0.021**	0.424	**0.004**
Year	0.026	0.855	0.034	0.334	0.026	0.680	0.033	0.344
Plot ID : Year	0.311	0.727	0.334	0.450	0.305	0.590	0.320	0.462
Residuals	0.230	NaN	0.232	NaN	0.219	NaN	0.222	NaN
**Leaves**^a^								
Plot ID	0.348	0.318	0.354	0.887	0.346	0.201	0.339	0.996
Year	0.082	**0.016**	0.043	0.179	0.121	**0.002**	0.051	0.087
Plot ID : Year	0.384	0.204	0.375	0.626	0.372	0.145	0.362	0.957
Residuals	0.186	NaN	0.228	NaN	0.161	NaN	0.248	NaN

^*^p-values < 0.05 are considered significant and highlighted in bold.

^a^Bark and leaves were only sampled at one timepoint, so this factor is excluded in these tests.

**Table 2:** Permutational analysis of variance (PERMANOVA) of the various beta diversity metrics (Bray Curtis, Jaccard, unweighted UniFrac, weighted UniFrac, Bray Curtis of k-mers, Jaccard of k-mers) of bacterial communities for the different vineyard sample types (berries, soil, bark and leaves)

	Bray Curtis		Jaccard		unweighted UniFrac		weighted UniFrac		k-mer: Bray Curtis		k-mer: Jaccard	
	R2	p-value^*^	R2	p-value^*^	R2	p-value^*^	R2	p-value^*^	R2	p-value^*^	R2	p-value^*^
**Soil**												
Plot ID	0.387	**0.001**	0.282	**0.001**	0.273	**0.003**	0.366	**0.013**	0.379	**0.012**	0.277	**0.001**
Time point	0.055	**0.001**	0.033	**0.002**	0.043	**0.001**	0.069	**0.005**	0.060	**0.007**	0.038	**0.001**
Year	0.022	0.203	0.025	0.019	0.031	**0.020**	0.036	0.082	0.027	0.188	0.030	**0.013**
Plot ID : Time point	0.186	0.780	0.216	0.939	0.207	0.961	0.175	0.845	0.171	0.944	0.211	0.976
Plot ID : Year	0.131	0.998	0.195	0.949	0.195	0.868	0.122	0.987	0.120	0.998	0.191	0.978
Time point : Year	0.015	0.763	0.020	0.651	0.021	0.457	0.014	0.683	0.014	0.803	0.019	0.802
Residuals	0.204	NaN	0.229	NaN	0.231	NaN	0.219	NaN	0.229	NaN	0.234	NaN
**Bark**												
Plot ID	0.420	0.390	0.437	0.157	0.434	0.212	0.387	0.220	0.415	0.387	0.435	0.304
Year	0.057	0.100	0.045	0.186	0.050	0.127	0.101	**0.008**	0.061	0.124	0.054	**0.043**
Plot ID : Year	0.364	0.238	0.353	0.138	0.362	0.165	0.407	0.086	0.373	0.262	0.345	0.367
Residuals	0.158	NaN	0.165	NaN	0.154	NaN	0.104	NaN	0.152	NaN	0.166	NaN

To assess potential confounding for within-group variability, Permutational Analysis of Multivariate Dispersions (PERMDISP) was calculated for each group and variable (see Supplementary Table 7).

^*^ p-values < 0.05 are considered significant and highlighted in bold.

Contrary to bark, the fungal communities on berries and leaves were less temporally stable and varied significantly between years (Table 1). Fungal communities of berries were most strongly affected by spatio-temporal factors. While the evenness is significantly different between the vineyards, the number of observed taxa also varied by time point and year (Supplementary Table 3). Fungal community composition varied substantially between vineyard, ripeness stages, and years (Table 1). When accounting for abundance, the explained variance nearly doubled. Interestingly, while these effects are robust across various diversity metrics and also significant when considered in interaction with each other (e.g. for plot and year, or year and time point), the overall explained variance is smaller in comparison to the other sample types (e.g. up to 16.1% explained by the vineyard and up to 2.7% explained by the year). Notably, both fungal communities of berries and leaves also exhibit significant within-group variability between years, as shown with Permutational Analysis of Multivariate Dispersions (PERMDISP) (Supplementary Table 6). This concurrent significance of PERMANOVA and PERMDISP suggests that part of the observed differences may be attributed to heterogeneity between timepoints. This is also reflected in the PCoA plots, which show both the dispersion within and the distinct clustering of each year (Supplementary Fig. 2).

Collectively, these results highlight the spatio-temporal variation in the vineyard microbial communities, with distinct patterns emerging for different ecological niches and microbial groups.

### Spatial structuring of fungal berry microbiomes

**Fig. 2:**
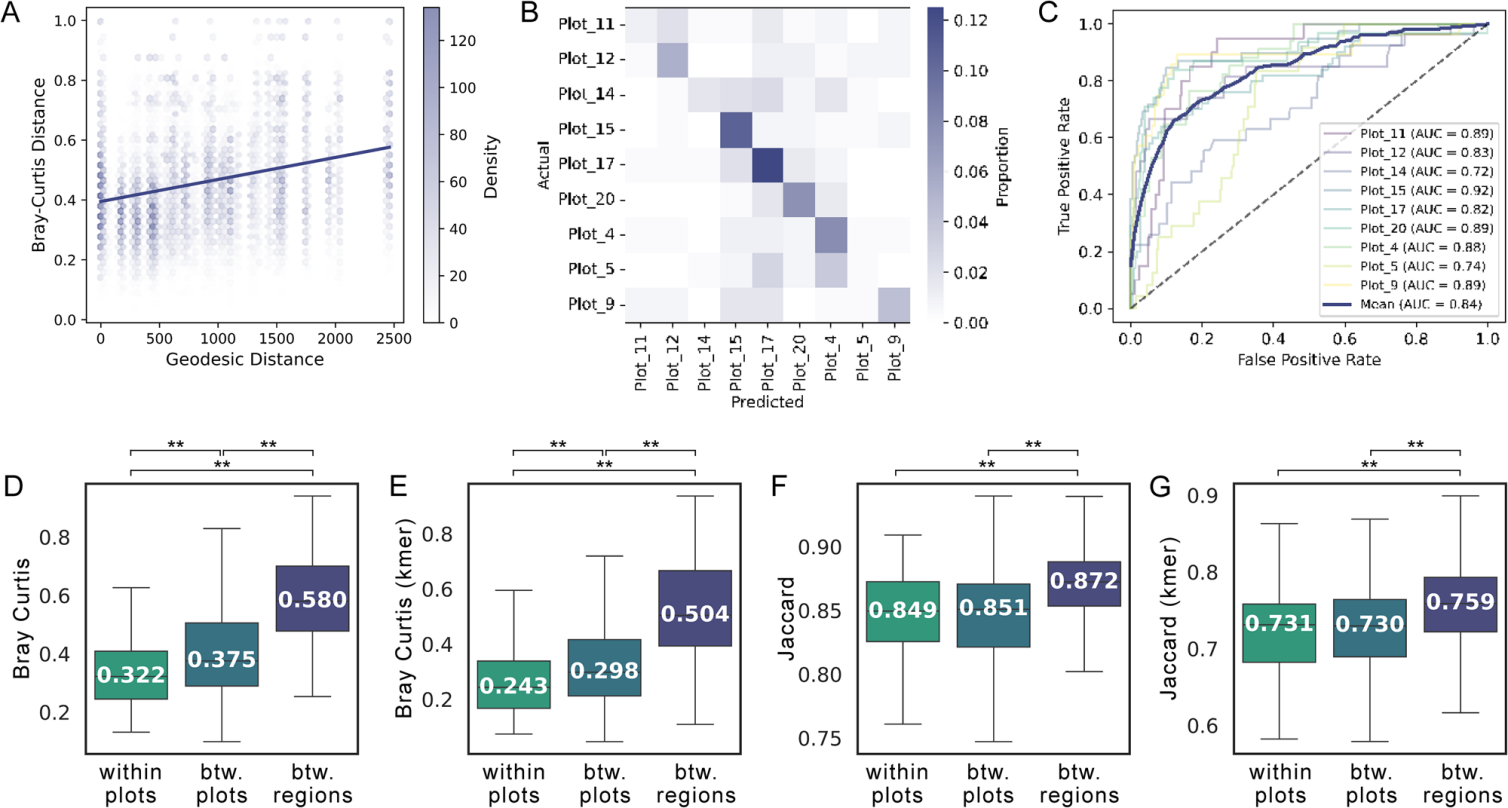
Berry fungal microbiome variation by site. **A** Vineyards within the same region exhibit a strong distance decay relationship, as shown by a Mantel test using the geodesic distance (in meters) against Bray-Curtis distance between berry fungal communities from the 2021 harvest. Prediction of site from fungal communities with a nested cross-validation random forest classifier (plots with fewer than 10 samples were excluded), as shown in the confusion matrix (**B**) and receiver operating characteristic (ROC) curves for the classifier performance (**C**). This is further reflected when comparing beta diversity distance within the same vineyard, or between vineyards of the same region or vineyards of different regions, using Bray Curtis (**D**), k-mer based Bray Curtis (**E**), Jaccard (**F**), and k-mer based Jaccard (**G**) metrics.

Beta-diversity analysis revealed site effects, which accounted for the largest proportion of the explained variation in berry fungal community structure within AOC Lavaux across different years, despite a maximum inter-plot distance of only 2.46 km. Notably, a significant heterogeneity in dispersion (see Supplementary Table 6) suggests that within-location variability contributes to these observed differences. The spatial imprinting was strong enough (and temporally robust) that it enabled successful prediction of the vineyard source using Random Forest machine-learning classifiers trained on fungal community composition from one year and testing it on a different year. The area under the curve (AUC) values, ranging from 0.62 to 0.76 (see Supplementary Table 8), reflected the classifier’s ability to distinguish between sites. This demonstrates moderate to strong predictive power in identifying the location based on the microbiome data between years, despite showing a high data-dependency and low generalizability. To further isolate the influence of this temporal variation we performed nested cross-validation random forest classification using only samples from harvest 2021, achieving a mean AUC of 0.84 (95% confidence interval: [0.67, 0.95]) (Fig. 2). Hence, microbial compositional similarity also appears to display temporal decay, with predictive accuracy of spatial location gradually decreasing from AUC of 0.84 within the same year (Fig. 2) to 0.74 (2022) and 0.62 (2023) in successive years (Supplementary Table 8). The overall high predictive accuracy underscored the plot-specific signatures on the fungal community composition, while highlighting the relationship of vineyard location and vintage.

To further understand the underlying spatial heterogeneity, we analyzed differences in fungal berry communities within vineyards as well as across regions. To capture intra-vineyard heterogeneity, we performed dense sampling in the AOC Lavaux vineyards at harvest in 2021, collecting samples approximately every 10th vine in every other row, excluding edge rows. To compare berry microbiota across regions, in addition to samples from AOC Lavaux, we collected samples from the adjacent AOC Valais of Chasselas and Pinot Noir vineyards at harvest in 2023 (see Supplementary Fig. 3). First, we compared various beta diversity metrics of the same grape variety from (i) within the same vineyards, (ii) vineyards within the same region and (iii) vineyards from different regions (Fig. 2). This showed that fungal communities from vineyards of different regions are robustly more dissimilar, than when comparing within the same region. This heterogeneity was more pronounced when taking the abundance of taxa into account. Using pseudo-phylogenetic metrics, i.e. considering the relatedness of present taxa, did not drastically change the results, indicating that the diversity between sites is not explained by differences in fungi belonging to entirely different clades but rather by closely related sub-species or strains.

Next, we evaluated the relationship between geographic proximity and community similarity. This revealed a robust distance-decay relationship in fungal berry microbiota, where spatially closer vineyards exhibited more similar fungal communities (Fig. 2, Bray-Curtis distance; Supplementary Table 9, all beta diversity metrics). This trend was observed even when comparing across vintages, indicating a strong and consistent influence of location on community assembly, despite significant inter-annual variations (Supplementary Table 9). While broad-scale (inter-vineyard) spatial effects were substantial, fine-scale (intra-vineyard) variation was generally weak (Supplementary Table 10). Only two vineyards, Plots 14 and 17, exhibited significant internal distance-decay relationships. While these plots do not stand out in terms of their size or topographical features, the surrounding land use and human activity might play a role in causing this internal spatial heterogeneity. Most of the sampled vineyards in AOC Lavaux are exclusively surrounded by other vineyards, but Plot 17 is flanked by a high-traffic road as well as a railway. While we are not able to conclusively determine the underlying reasons, it is noteworthy that the berry fungal microbiome can exhibit significant distance-dependent gradients even at small scales.

Additionally, spatial distance remained a significant predictor of fungal community composition even when comparing across different vintages or grapevine varieties (Supplementary Tables 9 and 11). This robust spatial signal underscored the dominant influence of location on community assembly, which is consistent with our PERMANOVA results (Table 1). While host genotype (variety) alone did not significantly affect fungal community composition, in the context of the sample location it showed robust effects across all metrics (Table 3). Notably, the within-group variability between varieties is significant for some metrics (see Supplementary Table 12), which calls for cautious interpretation. Nevertheless, this indicates that the host genotype effect is substantially smaller, and would be masked when not considering the sampling site.

**Table 3:** Permutational multivariate analysis of variance (PERMANOVA) with various beta diversity metrics for fungal communities of berry samples collected from AOC Lavaux as well as AOC Valais at harvest 2023

	Bray Curtis		Jaccard		k-mer: Bray Curtis		k-mer: Jaccard	
	R2	p-val	R2	p-val	R2	p-val	R2	p-val
Vineyard	0.523	**0.001**	0.370	**0.001**	0.619	**0.001**	0.410	**0.001**
Variety	0.007	0.219	0.007	0.916	0.005	0.278	0.005	0.970
Vineyard : Variety	0.109	**0.001**	0.086	0.781	0.108	**0.001**	0.072	0.988
Residuals	0.361	NaN	0.538	NaN	0.269	NaN	0.513	NaN

^*^ p-values < 0.05 are considered significant and highlighted in bold.

### Climate and topography shape berry microbiome assembly

With location having a dominant effect on fungal berry microbiome assembly, we further attempted to disentangle the influence of specific environmental factors by analyzing communities first in relation to individual variables, followed by a multivariate analysis to capture their combined influence. The vintage was the second most important factor in explaining variation in fungal communities (up to 2.7%, see Table 1). We therefore analyzed the climatic difference over the respective growing season for each year. Likely due to the close proximity of vineyards in Lavaux, we did not observe any significant differences in temperature or relative humidity when comparing within the same year (Supplementary Fig. 4). With climatic differences being more pronounced between years, their effect on berry-associated fungal communities is substantial. Relative humidity and temperature explained a variance of up to 4.2% and 0.6%, respectively (Supplementary Table 13, Supplementary Fig. 5). Yet, the specific climatic conditions of each plot, even subtle climatic variations, contributed to the observed biogeographic microbial patterns. This becomes apparent when considering the climatic influence nested per year, where relative humidity and temperature still significantly explain a variance of 2.7% and 0.6% respectively (Supplementary Table 14). Notably, differences in relative humidity had a stronger effect, and influenced the overall community structure of fungi, while the temperature solely had a significant effect on the presence or absence of fungi. K-mer-based metrics increased the explained variance, indicating that climatic conditions influence the community composition also on higher taxonomic levels. This is corroborated by the observation that fungal communities of vineyards with more similar climatic conditions also exhibit phylogenetically more similar fungal communities (Supplementary Table 15).

**Fig. 3:**
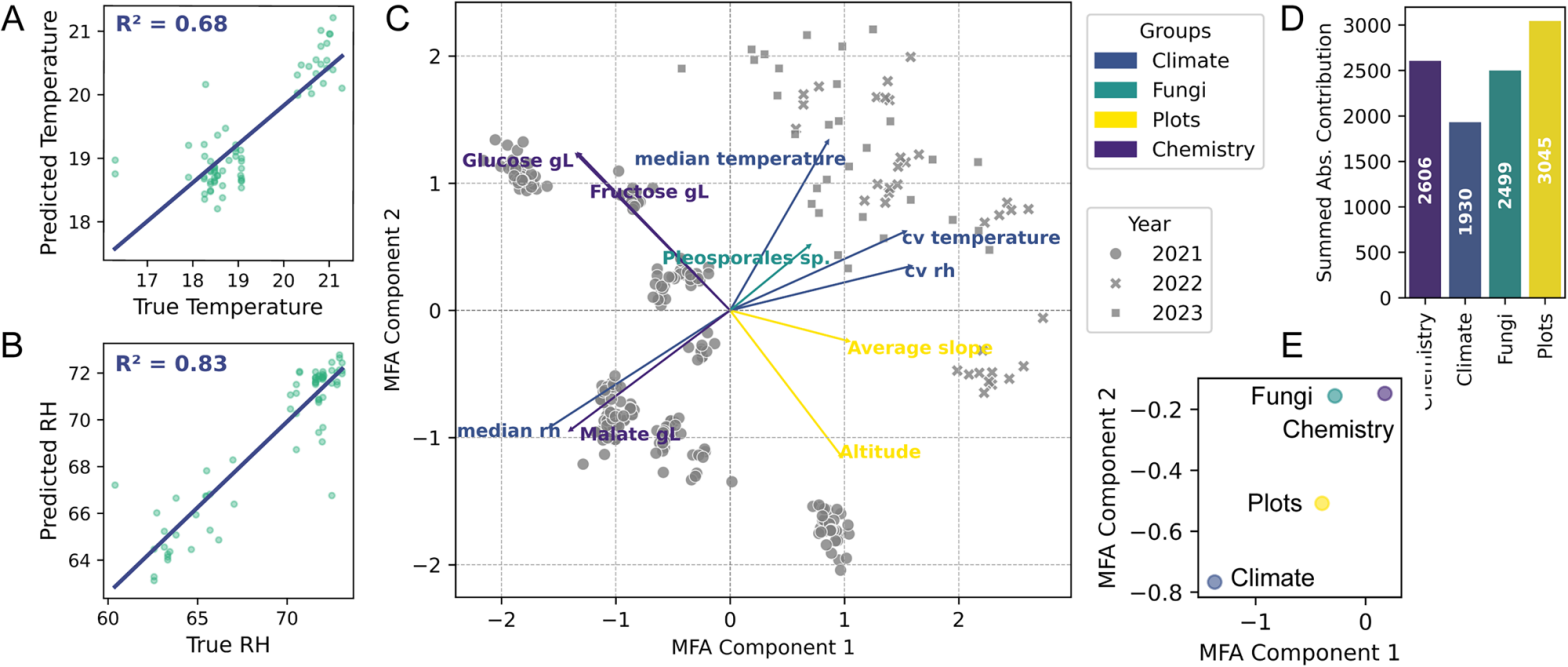
Microclimate prediction and multifactorial analysis of berry fungal communities. To predict the median temperature of the growing season, we applied a linear regression model (**A**) and a random forest regression model to predict median relative humidity (RH) (**B**). Multiple factor analysis (MFA) was used to disentangle the relationship between multiple data layers on the fungal microbiome of berries. Shown are biplots with the top 10 contributing features from all groups (**C**, colored by respective groups), as well the summed absolute contribution per group (**D**) and group representation (**E**).

Since different climatic conditions appeared to result in markedly distinct fungal communities, we attempted to predict the relative humidity and temperature from the community composition. Therefore, we used the machine-learning framework RITME (v1.0.4)^16^, which systematically optimizes feature transformation and model selection in relation to the target variable. We evaluated four distinct supervised learning models and an extensive hyperparameter search for model optimization (see Supplementary Methods and Supplementary Fig. 6). For predicting the temperature the best performing model was an elastic net linear regression, on ILR-transformed ASVs and data selection based on variance quantiles (above the 80th percentile), which explained 67% of the variance (Fig. 3). This may indicate that only a subset of fungal ASVs are responding strongly to slight changes in temperature. The optimized prediction of the relative humidity is derived from a random forest model with features aggregated on the family level and selected with a variance threshold (≥8.42e-05) without transformation, which explained 83% of variance (Fig. 3). This suggests a non-linear effect of humidity on fungal community composition and the influence on a higher taxonomic level and rather abundant or strongly co-occurring taxa.

To further study these complex interactions of key environmental and chemical factors influencing berry microbiome assembly, we conducted exploratory multiple factor analysis (MFA) integrating fungal microbiome data with plot topography, climate, and berry chemistry (sugars and organic acids). Despite the inherent complexity of the combined dataset, which explained only 18.56% of the variance with 10 components, the MFA revealed significant patterns. This suggests that much of the variation remains unexplained, likely due to unmeasured factors, interactions, or the inherent heterogeneity of microbiome data. The MFA clearly separated samples by vintage, with 2021 samples clustering distinctly, plausibly due to lower temperatures and humidity compared to 2022 and 2023 (Fig. 1, Supplementary Fig. 4). This is supported by the MFA biplot, where temperature and humidity metrics (median and coefficient of variation) strongly contribute to the first dimension (Fig. 3A). The second dimension is characterized by topographical features, highlighting how these factors collectively influence berry chemistry and fungal community structure, which are closely associated (Fig. 3E). While altitude and average slope were highly influential as individual factors, the combined contribution of all plot properties (including radiation and exposition) had the strongest overall influence on data structure (Fig. 3B). In correlation analysis, we previously observed robust associations between many of these factors, for example altitude and sugar content, which in the MFA appeared as opposing drivers of the data structure. This model thereby helps reveal the interconnection of various environmental factors in ultimately conveying a highly vineyard-specific imprint on fungal communities.

Overall, these results show that climate and topography collectively shape the dominant site-specific imprint on berry fungal communities and chemistry. Particularly, relative humidity emerged as a key factor influencing fungal community structure, affecting the presence of microbes and composition even on higher taxonomic levels.

### Environmental influences on soil microbial communities

The year, sampling time point, and location were all significantly associated with differences in the soil microbiota (Table 1 and Table 2). To better understand the influence of distinct environmental factors, we also performed MFA (Supplementary Fig. 7). In contrast to berry communities, soil microbial communities exhibited less interannual variability caused by climatic factors, which is consistent with the PERMANOVA results (Table 2). The plot topography, specifically the slope, appeared to be the most prominent contributing factor in shaping these vineyard soil microbiota. Regarding the soil properties, the interconnected variables of clay content and soil waterlogging (hydromorphy), as well as soil organic matter content and carbon-nitrogen ratio were key factors influencing communities. The even distribution of variance among groups in the MFA suggests that these factors are collectively crucial for understanding the observed microbial patterns. Furthermore, distinct bacterial clades showed significant variation between plots, with notable differences observed in the families *Steroidobacteraceae*, *Vicinamibacteraceae*, *Gemmataceae*, and *Micropepsaceae*.

### Fungal and fermentative yeast dynamics across environmental gradients

**Fig. 4:**
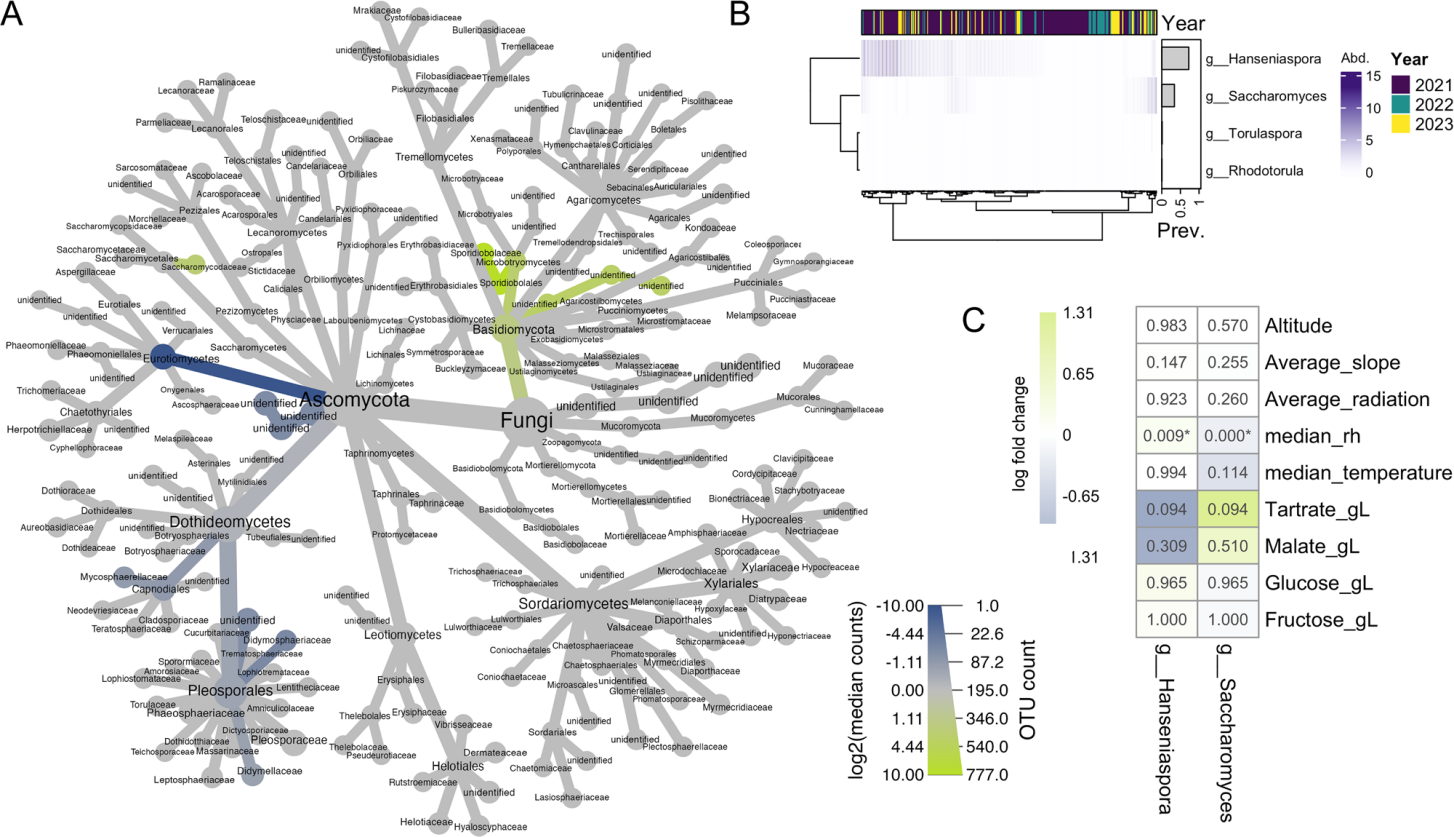
Environmental drivers of differential abundance in berry-associated fungal communities. **A** The berry associated fungal communities displayed at the family level, exhibit significant differential abundance between high and low relative humidity (for high relative humidity: enrichment in green, depletion in blue). Node size corresponds to overall feature count. **B** Log2-transformed abundance and overall prevalence of key fermentative fungal genera detected on berries at harvest across multiple years. **C** Differential abundance (ANCOM-BC2) analysis showing the log-fold change in bias-corrected abundance of *Hanseniaspora* spp. and *Saccharomyces* spp. associated with topographic, climatic, and berry chemical factors. Asterisks (*) denote statistically significant correlations (Holm corrected *p-val* < 0.05).

After demonstrating a robust association between environmental factors and berry mycobiome structure, we investigated the influence on specific taxonomic clades. Differential abundance analysis revealed associations between low and high relative humidity clusters with significant enrichment of *Sporidiobolaceae* and *Saccharomycodaceae*, while *Eurotiomycetes*, *Capnodiales*, and *Pleosporales* were depleted. Within *Saccharomycodaceae*, the fermentative yeast genus *Hanseniaspora* was specifically enriched under high relative humidity (Fig. 4).

Among yeast species involved in subsequent wine fermentations, *Hanseniaspora uvarum* exhibited the highest prevalence, being detected in 72% of all ripe berry samples. It is followed by *Saccharomyces cerevisiae* (33% prevalence), whereas *Rhodotorula babjevae* and *Torulaspora quercuum* were sparsely detected (Fig. 4). While most yeasts showed no significant inter-annual differences, *Hanseniaspora* spp. presence and abundance differed significantly between 2021 and 2022 (Fisher’s exact test for presence/absence, Bonferroni-corrected *p* = 0.0119; Wilcoxon Rank Sum test for log2-transformed data, Bonferroni-corrected *p* = 0.0117), suggesting that annual weather conditions could influence fermentative yeast prevalence and abundance in vineyards.

Next, we explored the influence of environmental factors and grape berry chemistry on the abundance of *Hanseniaspora* spp. and *Saccharomyces* spp. (Fig. 4). Both yeasts exhibit a strong, yet non-significant, correlation with berry chemistry. Interestingly, this is an inverse relationship, whereby *Saccharomyces* spp. abundance was positively associated with organic acids and *Hanseniaspora* spp. with sugar content and relative humidity. This is consistent with the original observation of enrichment of *Hanseniaspora* spp. in vineyards with higher relative humidity.

### Vintage and biotic interactions shape the wine metabolome

After identifying distinct microbial clades, particularly fermentative organisms, that varied with environmental conditions, we next investigated the relationship between vineyard properties, environmental factors, and the berry microbiome in shaping wine characteristics. Therefore, we conducted microvinifications of grapes from each plot over 3 years. To replicate typical wine-making conditions and to ensure consistency across years, we inoculated the must with a white-wine-specific commercial *S. cerevisiae* strain. To preserve vineyard-associated bacterial communities, we did not add sulfur dioxide.

As expected, the microbiota during early fermentation is characterized by high diversity and abundance of vineyard-resident bacteria and fungi, but eventually alpha diversity declines as fermentative yeast and bacteria dominate the fermentations (Supplementary Fig. 8). Beta diversity ordinations show a clear gradient in community composition throughout the fermentation (Supplementary Fig. 8). Notably, when considering solely the presence/absence of taxa, the ripe berries and must already exhibited distinct microbial compositions. This is likely due to the introduction of winery-associated microbes.

**Fig. 5:**
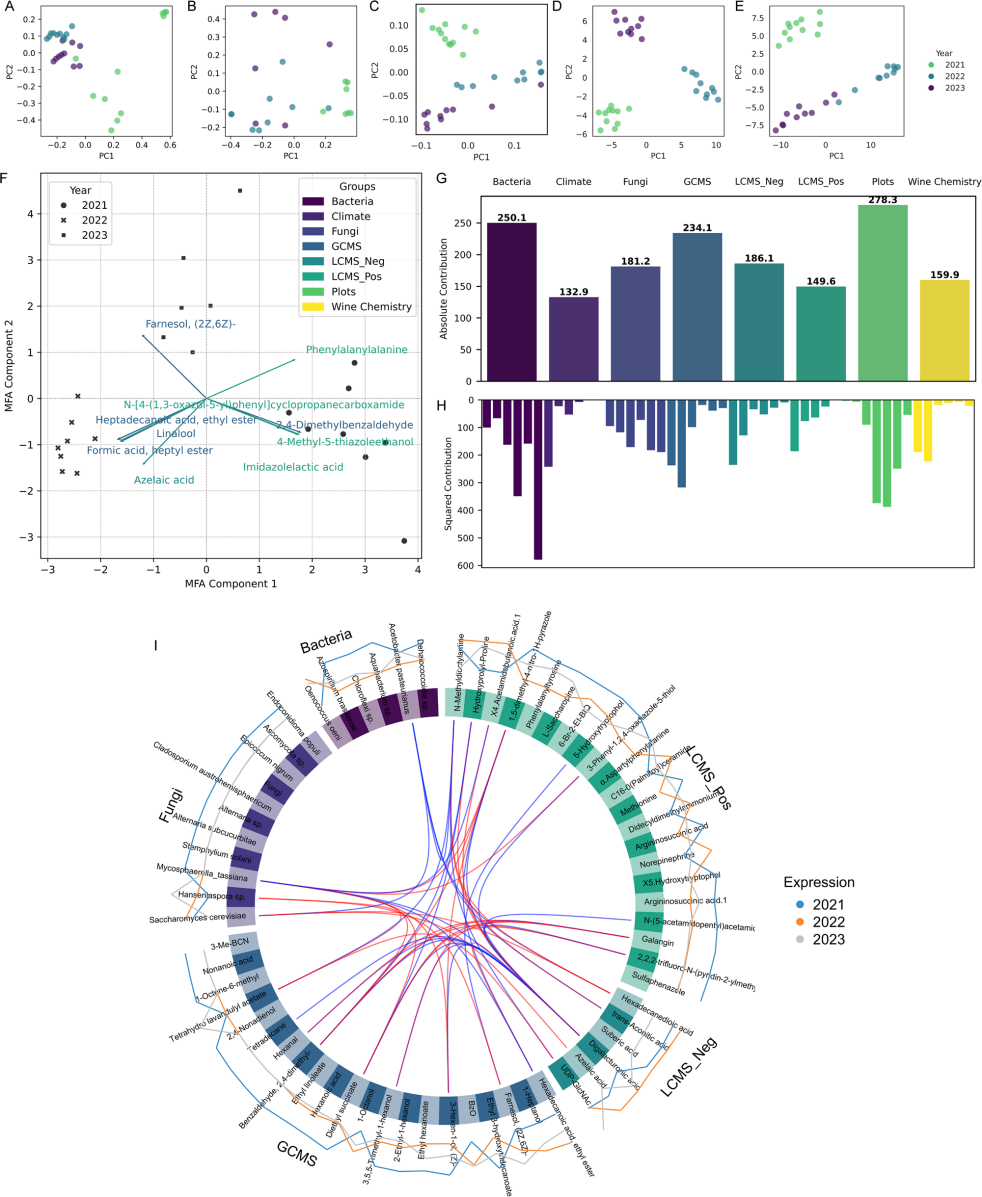
Ordination plots and multifactor analysis (MFA) to compare wine samples across different years with the various multi-omics data layers. **A** PCoA plot for Jaccard metrics for fungal communities and **B** Jaccard metrics for bacteria, **C** PCA plot of head-space gas chromatography-mass spectrometry (HS-GC-MS) data, **D** PCA plot for negative mode liquid chromatography-mass spectrometry (LC-MS) and **E** PCA plot for positive mode LC-MS. Exploratory MFA of wine samples with microbiome (fungal and bacteria), untargeted metabolomics (GC-MS, LC-MS), wine chemistry, climate data and topographical vineyard properties. **F** Biplot of top 10 contributing features in the first 2 dimensions as well as **G** the absolute summed contributions per group and **H** per dimension. Additionally, we performed a supervised, horizontal integration Partial Least Squares Discriminant Analysis (PLS-DA) model on the microbiome and metabolome data, with **I** showing a circos plot of top correlations (cut off at 0.90, positive correlations in red and negative correlations in blue).

To further explore the relationship between microbial communities and wine characteristics, we performed untargeted analysis of wine metabolites using three distinct methods: head-space gas chromatography-mass spectrometry (HS-GC-MS) for volatile compounds, and liquid chromatography-mass spectrometry (LC-MS) in both positive and negative ionization modes. The integration of these multi-omics data layers aims at providing a comprehensive framework for understanding the spatio-temporal influences on wine characteristics. Ordination analyses consistently revealed a clear clustering of wine samples by vintage across all data layers (Fig. 5). This observed temporal stratification underscores the substantial impact of inter-annual variations on the microbial and metabolic composition of the wines, and reflects the dynamic nature of viticulture and winemaking (Fig. 5).

To further study the interactions between multi-omics layers, vineyard topography, key wine chemistry parameters, and climatic factors, we conducted MFA using six components that explained 63.12% of the variance. The ordination plot of the first two components, accounting for 21.9% and 12.0% of the variation, respectively, show a clear clustering by year, with various metabolites identified through GC-MS and LC-MS analysis driving the spread (Fig. 5). When considering the absolute group contributions, the topographic properties of the vineyards had the greatest influence on the overall data structure. Notably, this contribution is most pronounced in the 3rd and 4th dimensions of the model. In contrast, climate had the strongest influence on the first dimension. This may explain the horizontal spread observed between the samples from 2021 and 2022/2023, as these years had the largest climatic differences. These climatic variations are linked to corresponding differences in metabolites and wine chemistry, as these groups also are prominently associated with the first two dimensions of the model. Fungal and bacterial communities, however, show a more complex relationship to the other datalayers, as their group contributions are substantial across all dimensions. This suggests that these microbiomes are influenced by a combination of factors, including topography, the climate, as well as resulting wine chemistry and metabolites, which collectively shape their community structure.

While the unsupervised MFA revealed broad trends, we next applied a multiblock, supervised Partial Least Squares Discriminant Analysis (PLS-DA), specifically a Data Integration Analysis for Biomarker Discovery using a Latent Variable Approach for Omics studies (DIABLO), with two components. This approach allowed us to mine microbiome and metabolomics data for key discriminatory features and gain mechanistic insights. Using year as the target variable, we sought biomarkers that explain vintage-related differences. The variance between years was evenly distributed between the two model components (51.7% and 48.2%), confirming the robustness of the model in capturing group differences across the integrated datasets. PLS scores plots (Supplementary Fig. 9) show strong clustering of each data layer by year, with more overlap in bacterial communities. The variable plot (Supplementary Fig. 9) highlights the most influential features driving the dispersion across components and their correlations. Most metabolites and microbes cluster to one side, with several key features standing out in driving the differences between years. Notably, we observed *Hanseniaspora sp*. and *S. cerevisiae*, each closely associated with various metabolites forming distinct clusters. This is also further illustrated in the circus plot, showing the top correlations and potential biological interactions among features (Supplementary Table 16).

Several intriguing correlations between the microbiome composition and wine metabolome indicate bi-directional interactions (Fig. 5). A strong positive correlation was observed between *S. cerevisiae* and LC-MS-detected acids, such as the likely plant-derived dicarboxylic hexadecanedioic acid and azelaic acid, which is known to have antibacterial properties. This is consistent with a strong negative correlation between *Acetobacter pasteurianus* and *S. cerevisiae*, suggesting that plant metabolites, which suppress bacteria, may indirectly promote yeast growth. *Hanseniaspora sp*. was strongly associated with volatile compounds like Farnesol and Tetrahydrolinalyl acetate (Tetrahydro lavandulyl acetate), which are both floral scents. Interestingly, the model also revealed strong positive and negative correlations of various metabolites, among them diethyl succinate which plays a role in the fruity aroma profile of wines, with the common grapevine endophytic fungus and opportunistic pathogen *Mycosphaerella tassiana*. We also observe multiple correlations among metabolites involved in the plant stress response, many of which are aromatic compounds, such as Hexanal, Farnesol, Tetrahydrolinalyl acetate, and Diethyl succinate (Supplementary Table 16).

In summary, by integrating environmental and multi-omics data, we highlight the complex interactions that shape the berry microbiome and metabolome. The analysis of specific interactions between bacteria, fungi, and metabolites sheds light on the plant’s biotic stress response, the interplay between microbes, and the direct link between fungal species and aroma formation in wine fermentation.

### Multifactorial analysis of wine sensory profiles

**Fig. 6:**
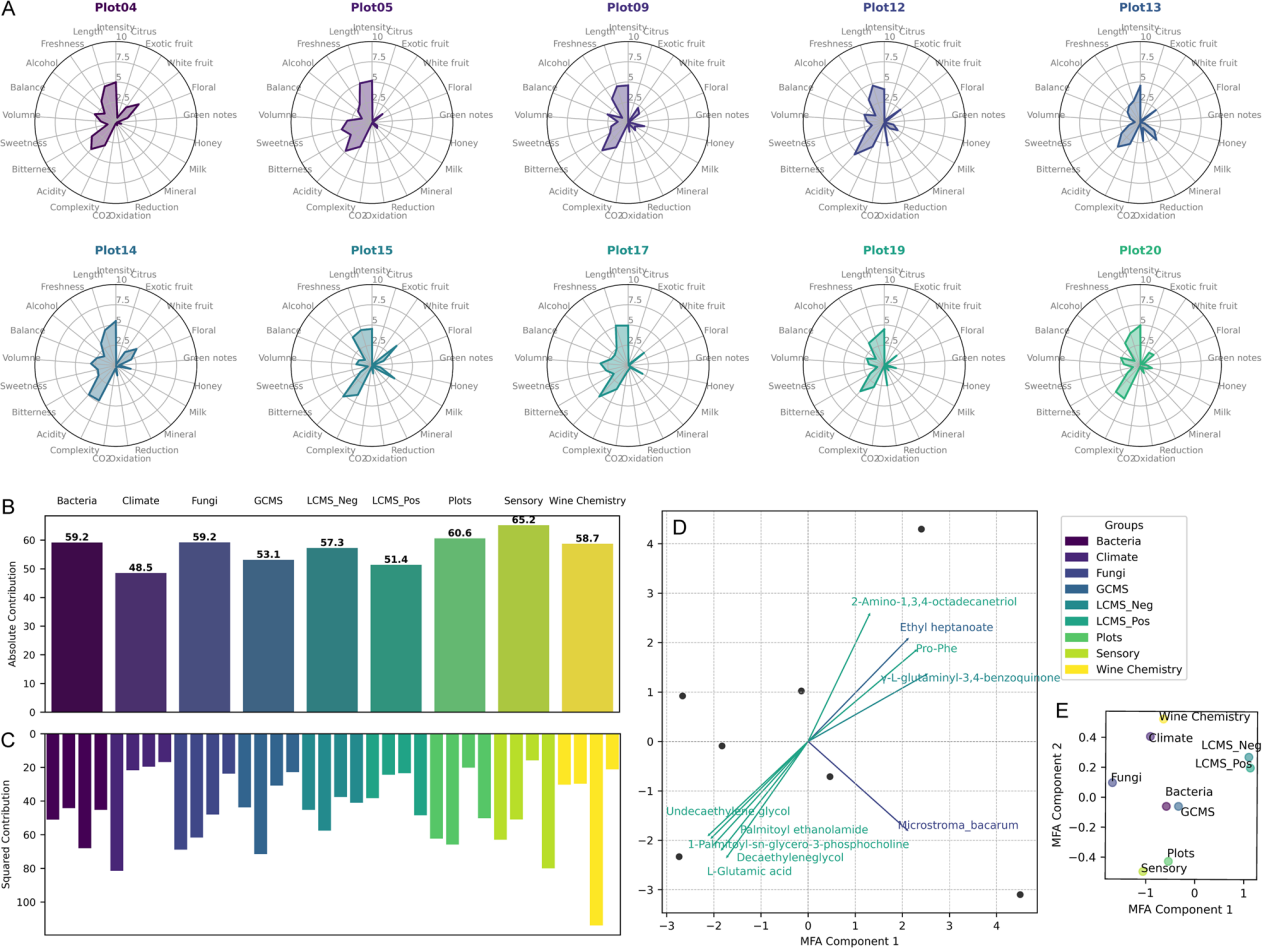
Integrative analysis of multi-omics and sensory data. **A** Sensory spider plots illustrate the distinct characteristics of wines produced from different vineyards. **B** Multifactor analysis (MFA) results show the summed contribution of each group to the model, as well as **C** contributions of each group per dimension. **D** The biplot with top 10 loadings reveals the association between specific metabolites and olfactory features, highlighting their role in driving the dispersion of wine samples. **E** Group representation shows the clustering of groups, highlighting their close interconnectedness.

Having shown the strong correlation between fermentative yeast species and volatile aromatic compounds, we further explored olfactory differences in wine characteristics through sensory analysis. A panel of experts evaluated each wine based on a set of predefined criteria typical of Chasselas wines from AOC Lavaux. Although there were substantial and significant differences between assessments (see Supplementary Fig. 10), the wines from different vineyards still showed distinct flavor profiles (Fig. 6). We integrated environmental factors, microbiome and metabolome data from the respective year as well as the sensory data using MFA, with a strong fit (four components explaining 77.19% of the variance). The contributions of the various groups were relatively balanced. Climate overall showed the smallest, yet most distinct effect on the first dimension. This aligns with previous observations that climatic differences between years were more significant than those within a single year within the AOC region. The biplot of top loadings further highlights the strong correlation between specific metabolites, most of which were measured in the LC-MS positive ionization mode.

Further investigating these correlations revealed strong associations between topography, climate and sensory properties. Specifically, higher altitude is strongly associated with lower median temperature (Spearman correlation strength = −0.96, *p* = 0.0005) and higher temperature variability (corr = 0.89, *p* = 0.0068), which in turn appear to influence sensory properties. Warmer and more stable climates enhance volume in the mouth (corr = 0.86, *p* = 0.0137), aromatic length (corr = 0.93, *p* = 0.0025), acidity (corr = 0.93, *p* = 0.0025), and sweetness (corr = 0.86, *p* = 0.0137), while greater temperature variability reduces these attributes (corr = −0.86 to −0.82, *p* < 0.025). Southwest-facing exposure was associated with reduced green notes (corr = −0.79, *p* = 0.034) and olfactory complexity (corr = −0.79, *p* = 0.034), likely due to increased sun exposure promoting ripeness. Notably, the two sensory characteristics, exotic fruit and balance, are not significantly associated with any climatic features, topography, or other fungal taxa, but exclusively with the fermentative yeast *Hanseniaspora spp*. (corr = −0.82 and −0.81, *p*-values = 0.023 and 0.027, respectively). *Mycosphaerella tassiana*, and other putative pathogenic fungi (*Alternaria spp, Stemphylium solani, Ustilago hordeii, Cryptovalsa ampelina*, *Endoconidioma populi, Curvularia trifolii*) were shown to strongly positively correlate with the sensory perception of Oxidation (corr = 0.75 to 0.91, *p* < 0.05), but commonly had an equally strong negative correlation with Olfactory Intensity (corr = −0.75 to −0.81, *p* < 0.05) in resulting wines.

## Discussion

The concept of *microbial terroir* describes the influence of site-specific microbial communities on distinct product characteristics and has been reported for a variety of foods^17–19^, but plays a particularly prominent role in viticulture^5,20–22^. Microbial communities in vineyards influence plant health^23^ and berry qualities, as well as contribute directly to subsequent fermentation^4,14^. Although vineyard location is commonly reported as a primary factor differentiating grapevine-associated microbiomes^1^, the specific environmental factors involved, their interactions, and their impacts on resulting wine characteristics remain poorly understood.

Our study provides a more granular and comprehensive perspective by disentangling location-dependent environmental factors within a tightly controlled and spatially linked experimental framework that controls for biotic factors. This allows us to dissect associations between specific environmental factors and grapevine- and wine-associated microbiota and metabolomes. Even within a small geographic region, we observed highly distinct microbial communities, shaped by site characteristics, annual and seasonal fluctuations, and microclimatic conditions, which reflect general ecological principles of environmental filtering and microbial niche differentiation.

Soil is a fundamental element in *terroir*, providing the vine with water and nutrients, and in the context of *microbial terroir*, acting as a primary microbial reservoir. It serves as a key source for the colonization of annually growing leaves and berries^12,24^, with microbial transmission occurring through various potential vectors^1^. In our study, soil microbial communities exhibited strong spatial structuring, forming the foundation for subsequent heterogeneity in other vineyard-associated microbiomes. At the intra-region scale (max 2.46 km distance between vineyards) soil microbiota variation was not structured by geographic proximity, as was reported in studies across larger distances^25^. This is particularly interesting, as in berries we observe a strong distance decay relationship, indicating distinct transmission, dispersion and environmental filtering mechanisms for these different microbial habitats. Microbial diversity in soil was affected by intricate interactions between inherent soil properties, such as clay content and connected waterlogging, as well as dynamic factors like soil carbon and nitrogen levels. These factors all contributed to observed seasonal fluctuations, which was also reflected in soil pH—a key variable which is influenced by climate, altering microbial activity and thereby the organic matter content^26^. In contrast, microbes colonizing aboveground plant organs are exposed to harsh conditions, such as UV radiation and fluctuations in temperature and humidity^1^, imposing strong selection pressures and shaping the composition of epiphytic communities.

As such, and most similar in richness and community composition to soil, bark is also a key microbial reservoir. Fungal communities of bark showed significant spatial differences, which were stable across the years. This may be connected to long-term niche adaptation, as fungi form stable communities in the rough surface of bark, which become more complex as the plant ages^27^, as well as the dispersal limitation of fungi leading to more spatial heterogeneity^20^. Conversely, bacterial communities on bark varied more strongly between years than between sites, indicating a more dynamic response to climatic fluctuations.

Fungal communities on leaves and berries, as perennially colonized organs, are as expected variable between the years. Whereas the berry microbiota before ripening closely resembles that of leaves^28^, the accumulation of sugars and other metabolites leads to a niche specialization in later stages^1^. Interestingly, while leaf microbiota were not significantly different between vineyards, the berry-associated fungal communities exhibited the strongest spatio-temporal partitioning of all sample types. The site-specific imprint was notably strong, with some vineyards even exhibiting significant internal heterogeneity. Predictions of the vineyard location from the fungal berry microbiota improved when controlling for host genotype or vintage, or comparing across larger geographic scales. This highlights the mutual influence of host genotype and site-specific factors on community assembly. The site-specific factors associated with diversity of berry fungal microbiota are primarily attributed to climatic and topographic variation between vineyards. Larger inter-annual climatic differences had a corresponding larger influence. Yet even insignificant microclimatic differences between plots exerted a significant effect on fungal communities, with climatically more similar plots also harboring more similar fungal communities. Relative humidity exhibited particularly strong effects on taxonomic community structure, including a noteworthy association with the fermentative yeasts *Hansensiaspora spp*. and *Saccharomyces cerevisiae*. Notably, this appears to be an inverse relationship, with climatic factors and resulting berry chemistry either favoring *Hansensiaspora spp*. or *Saccharomyces cerevisiae*, which also showed to be a key distinction between wines of different years.

These observed microbiome variations between plots and years were further reflected in distinct metabolomics profiles of the resulting wines. Inter-annual climatic variations as well as fine-scale site-specific differences contributed to distinct characteristics. We found that *Hanseniaspora spp*. are strongly associated with the expression of floral aroma compounds across vintages, and distinctly correlated with exotic fruit aroma and balance of samples from the same year. Previous lab-controlled studies^29,30^ have shown that fermentation with *Hanseniaspora* species contributes to more fruity and floral aromas, with species and strain variability playing a significant role in the formation of specific aroma compounds^31^. *Hanseniaspora* spp. have therefore been suggested to be major contributors to *terroir* expression. These yeasts are particularly influential during the early stages of fermentation, as they are more alcohol-sensitive, underscoring the importance of fermentation practices on their aroma-producing potential^22^. Interestingly, the prevalence of *Hanseniaspora* species has been shown to vary between grape varieties^32^, regions^22,33^, vineyards^2^, years^34^ and with berry ripening and health status^28^. We contribute to this, by providing a promising, direct link between site-specific climatic factors, microbial differences, and fermentation metabolites, leading to sensory variations in resulting wine.

Notably, when comparing fermentation dynamics across different years, we observed a significant interaction between plant stress response metabolites, microbial communities and aroma formation. Specifically, higher levels of azelaic acid, a key signaling molecule involved in the expression of antimicrobial genes as part of the plant’s pathogen stress response^35^, were associated with increased growth of *Saccharomyces cerevisiae*. Azelaic acid serves as a pathogen response signal and plays a role in lipid signaling related to grapevine defense against downy mildew^35^ and esca disease^36^. Such associations between grape volatiles, lipid metabolism and the associated microbiome have previously been suggested^37^. Similarly, we observed multiple correlations among metabolites involved in plant stress response, many of which are aromatic compounds. Microbes, including yeasts and potential pathogens, have been shown to modify plant volatile compounds in grapevines^38^ and our findings highlight these interactions between microbes and plant biotic defense mechanisms contributing to wine aroma formation.

Additionally, our results highlight an important but often overlooked aspect of *microbial terroir*: the impact of vintage. While it is well understood in winemaking that vintage influences wine quality, there is a common misconception that microbial communities should remain stable if they are truly regional. In reality, these communities are shaped not only by location but also by annually variable environmental conditions. As we show, 2021 differed markedly from 2022 and 2023, which experienced more similar weather patterns. Vineyard microbiomes—and the resulting wine characteristics—respond to these fluctuations and we observed a form of temporal decay, where microbiota from consecutive vintages tend to be more alike. Recognizing *microbial terroir* as a dynamic expression of place and time requires that future studies of food system microbiomes consider not only spatial but also temporal variation. Particularly when studying plant-microbe interactions or fermentations, strain diversity plays a crucial role, and within-species variation can influence whether a microbe contributes to aroma formation or spoilage, or acts beneficial or pathogenic^39^. Our analysis was conducted primarily on the level of amplicon sequencing variants and is limited in the taxonomic resolution, as well as being constrained to relative and compositional data^40^. Although the large number of samples provides robust insights, due to the resolution afforded by marker-gene amplicon sequencing we cannot track the transmission of specific strains between plant organs, fermentation stages, and across time and space.

Studying microbial communities in an open system also comes with additional inherent limitations, including unmeasured influence factors that may potentially influence measurements, such as microclimatic effects or microbial dispersal through human activity during harvest or traffic^1^. Additionally, stochastic effects of ecological drift and randomness in microbiome assembly likely contribute to observed variations in community composition^41^. Importantly, our analysis is based on associations across data sets, which can provide strong support for our hypothesis but cannot definitively establish causal relationships. Another strength, yet in this context a limitation of our study, is the setting in commercially managed vineyards. While this conveyed insights into microbial diversity in real-life settings, we could not control the application of fungicides and herbicides as well as under-vine management practices. Showing the connection between environmental factors, plant stress, associated microbes and resulting wine characteristics therefore calls for future studies systematically exploring the interactions with vineyard management, in particular in relation to abiotic plant stress. Despite these challenges, the overall study is strengthened by a relatively well-controlled setup and multiple biological replicates across locations and time points, as well as the use of complementary analysis approaches. While environmental gradients and associated community dynamics will expectedly differ between regions, the consistency of our findings across sites and years supports the robustness of the observed patterns and associations.

Overall, our study provides insights into complex microbial dynamics in vineyards and subsequent fermentation, and successfully disentangles abiotic contributions in highly interconnected systems. We advance the understanding of *microbial terroir* in viticulture and winemaking by showing how fine-scale environmental variation shapes microbial diversity and affects interactions between the microbiome and metabolome of grapevines and wine. Although focused on viticulture, the observed patterns reflect broader ecological principles of how fine-scale environmental variations shape microbial assembly and function in plant microbiomes. However, establishing causal relationships among these factors remains a key challenge. The *microbial terroir* research community must also recognize the need to control many covariates for optimal study design, and address the challenge of controlling these factors in commercial vineyards. Future research will require higher-resolution sequencing technologies, larger sample sizes, and higher temporal resolution to track transmission of strains and metabolomics profiling to fully reveal these intricate interactions.

## Methods

### Longitudinal sampling and environmental data collection in AOC Lavaux

In the AOC Lavaux subregion Villette, we collected samples and data over 3 years (2021–2023) from 12 different vineyards, two of which had their vines uprooted within this time span. The vineyards are all within a 2.5 km radius and were selected to represent maximal pedoclimatic variability present in the AOC region. They are uniformly planted with *Vitis vinifera* clonal variety Chasselas on a 3309C rootstock. Vine age ranges from 15 to 25 years and all vines are guyot-pruned and trained in a vertical shoot positioning system^15^. Over the growing seasons, we recorded vineyard management and vine development, i.e. application of herbicide, hail damage, mildew appearance, estimated grape yield and pruning weight, as well as records of phenological stages of budburst (BBCH 09) and flowering (BBCH 65) on 100 randomly selected plants within a plot^42^.

To record climate data, we installed sensors (HOBO U23 Pro v2 Temperature/Relative Humidity Data Logger from Onset, Cat No. U23-001A) with radiation shields (Onset, Cat No. RS3-B) for improved temperature measurements at approximately 1.8 m height within each vineyard plot. These continuously measure the temperature and relative humidity every hour for 3 years. We removed outliers of the climate data (generated by e.g., dropped probes, empty batteries) with rolling Median Absolute Deviation (MAD) and subsequently calculated the daily median values over each growing season (1 April to 30 September). Additionally, we calculated the Growing Degree Days (GDD) for each plot and year.

Samples for microbiome analysis were collected over a three-year period. Rhizosphere samples were taken twice per year (in spring and at veraison), while bark and leaf samples were collected annually at veraison. Berry samples were collected twice per year, at veraison and harvest. From the Valais vineyards we collected berries in 2023 at harvest. To collect rhizosphere samples, the surface layer of soil (~3 cm) was removed and three soil cores were then taken in proximity to the stem (within 20 cm from the trunk) to 30 cm depth. Soil samples were pooled and sieved with a 1 mm mesh size to remove stones. For grape sampling, clusters of berries were cut off using sterile scissors. Bark and leaf samples were also taken using sterile scissors, ensuring no contamination occurred during the sampling process. All collected samples were transported on ice back to the laboratory and stored at −80 °C until further processing.

### Berry chemistry

To assess berry maturity we used high pressure liquid chromatography (HPLC) to analyze the concentrations of glucose, fructose, malic- and tartaric acid in grape must samples^43^. Glucose and fructose are essential for fermentation of grape juice to wine, and are an indicator of berry ripeness. Tartrate and malate were investigated because they are the most prominent organic acids in wine, making up 90% of total acidity in grape juice^44^. Therefore, we used diluted 500 µL (1:5, with ultrapure Milli-Q water) and filtered (0.2 μm) grape must in HPLC closed glass vials (e.g. crimp neck from BGB Analytik AG, Cat No. 0800035 and alu crimp caps with septum from BGB Analytik AG, Cat. No. 080304) and put them in an autosampler (Merck Hitachi, No. L-7250). To calculate the concentration of each component, a calibration curve with solutions of the named sugars and organic acids was used. All standards, e.g. D-(+)-Glucose 99.9% (Cat No. PHR1000), D-(−)-Fructose ≥99% (Cat No. F0127), DL-Malic acid ≥99% (Cat No. 240176), L-(+)-Tartaric acid ≥99.5% (Cat No. T109), were purchased from Sigma Aldrich. Samples were run on a HPLC-UV-RI Agilent 1260 Infinity with a carbohydrate column HPLC Aminex HPX-87H (300 ×7.8 mm) column (Biorad, Cat. No. 1250140) and a guard column e.g., Cartridges Carbo-H (4 ×3.0 mm) (Phenomenex, Cat No. 125-0129). The column is heated to 35 °C (column oven from Merck Hitachi, No. L-7300), 20 μL of the samples is injected into the system and eluted with 0.65 mM H2SO4 at a flow rate of 0.5 mL/min (pump from Merck Hitachi, No. L-7100). UV detection is carried out at 210 nm and analysis in the EZChrom Elite software (Agilent).

Further, we measured the Brix value of berries using a handheld refractometer for sugar (VWR Cat. No. 635-0631, 0–32% Brix, 0,2 °Brix resolution). To assess vine water status and photosynthetic carbon isotope composition as a proxy for vine water stress, the 12 C/13 C ratio (δ13C) in sugars of ripe berries was analyzed in 2023 by Laboratories Dubernet in Narbonne, France. Additionally, yeast-assimilable nitrogen (YAN) was measured by Fourier Transform Infrared Spectroscopy (FTIR) at Changins.

### Soil analysis

To measure the pH of each soil sample, we followed the Standard Operating Procedure by the FAO^45^. In short, we used 0.35 g of soil with 1.75 µL of 0.01 M CaCl2 (Sigma Aldrich, Cat No. 1.02382) solution in a Deep Well plate, which is homogenized for 30 min at 8 Hz in a TissueLyser (QIAGEN) before let settle for another 30 min and measured with a calibrated pH meter.

The Sol-Conseil laboratory in Changins analyzed soil samples collected from 10 plots in 2023. Samples were conditioned (dried for 48 h at 40 °C and sieved by 2 mm) and subsequently analyzed for total nitrogen content, and C:N ratio as well as particle size distribution [Sand/Silt/Clay], including total organic matter (granulometrie).

### Sample processing and DNA extraction

For DNA extraction, the grapes were thawed and pressed using a stomacher. A 250 µL portion of the homogenized grape material was used for DNA extraction using the MagAttract PowerSoil Pro DNA Kit (QIAGEN, cat. no. 47109) and the PowerBead Pro Plate (QIAGEN, cat. no. 19311), following the manufacturer’s instructions. Cell lysis was performed using a Bead Mill (Retsch), and DNA extraction was carried out on a KingFisher Flex (Thermo Fisher Scientific). The extracted DNA was stored at −20 °C until further processing.

### Library preparation and sequencing

Using the HighALPS ultra-high throughput library preparation protocol^46^, which based on a unique dual index (UDI) strategy with custom 12 nt long Golay barcodes, we profiled present bacterial communities with amplicon sequencing of the hypervariable V4 region of the 16S rRNA gene using the updated 515 F primer (5’-GTGYCAGCMGCCGCGGTAA-3’)^47^ and 806 R primer (5’-GGACTACNVGGGTWTCTAAT-3’)^48^. For fungal communities, we amplified the first loci in the internal transcribed spacer (ITS) region with above-described BITS and B58S3 primers^49^. To amplify the low microbial biomass samples we performed a nested PCR. In the first PCR, all template DNA is enriched with 515F/806R primers and in the second PCR, the amplicons are UDI barcoded as well as plant host plastid and mitochondrial 16S rRNA gene contaminations depleted with peptide nucleic acid (PNA) PCR clamps^50,51^. Therefore, we first set up a 25 µL PCR reaction with 12.5 µL of 2x KAPA HiFi HotStart ReadyMix (Roche, Cat. No. 07958935001), 0.4 µM of each primer (Microsynth) and 2.5 µL of extracted DNA. All PCR reactions were set up using an epMotion liquid handling platform. Reaction conditions were initially 95 °C for 5 min, followed by 35 cycles of 95 °C for 20 s, 49 °C (ITS)/55 °C (16S) for 15 s, and 72 °C for 15 s, and a final extension of 72 °C for 5 min. From this enriched template, we used 1 µL per 25 µL reaction for a second PCR with 12.5 µL of 2x KAPA HiFi HotStart ReadyMix, 0.4 µM of UDI primer combinations as well as 0.5 µM or mPNA and pPNA clamps. The PNAs were vortexed and incubated at 60 °C for 10 min before use. The subsequent reaction conditions were 95 °C for 5 min, followed by 8 cycles (ITS) and 12 cycles (16S) of 95 °C for 30 s, 78 °C for 5 s for PNA annealing (only for 16S), 49 °C (ITS)/50 °C (16S) for 20 s, and 72 °C for 15 s, and a final extension of 72 °C for 5 min. Notably, the presence of PNAs in the reaction showed that a lower annealing temperature increased yield. Resulting DNA concentrations were measured in duplicates with Qubit dsDNA High Sensitivity Assay (Thermo Fisher Scientific, Cat No. Q32854) on a Tecan Spark Microplate Reader. Amplicons were pooled equimolarity in two separate pools with a liquid handling platform (Brand GmbH, Wertheim, Germany) and purified with Agencourt AMPure XP magnetic beads (Beckman, Cat No. A63882) with a ratio of 0.7X to remove primer dimers and small fragments on the KingFisher Apex. Quality of the resulting amplicons was controlled on a Tapestation (Agilent). To increase yield we reconditioned each pool separately with 12.5 µL of 2x KAPA HiFi HotStart ReadyMix, 0.4 µM of the standard Illumina P5 (5′-AATGATACGGCGACCACCGAGATCT-3′) and P7 (5′-CAAGCAGAAGACGGCATACGAGAT-3′) PCR primers and 2 µL template of the bacterial pool and 3 µL template of the fungal pool. Reconditioning PCR conditions were set to initial 95 °C for 3 min, followed by 4 cycles of 98 °C for 20 s, 62 °C for 15 s, and 72 °C for 30 s, and a final extension of 72 °C for 1 min. Ultimately, we conducted a two-sided clean up with 0.2X magnetic bead ratio, followed by 0.7X magnetic bead ratio of the supernatant to remove remaining genomic DNA as well as primers from the reconditioning. The pools were combined and submitted to the Functional Genomics Center Zürich for Paired-End 250 bp sequencing on the Illumina NextSeq 2000 P1 600 cycles with 20% PhiX.

### Microbiome data analysis

Microbial diversity analysis was performed with QIIME 2 (version 2024.10)^52^. Demultiplexed Illumina FASTQ files were imported, and residual adapter sequences were removed with cutadapt^53^. Amplicon sequence variants (ASVs) were generated by denoising single-end reads with DADA2^54^. Taxonomic classification of ASVs was performed with the q2-feature-classifier plugin^55^. For 16S rRNA gene ASVs, a naive Bayes classifier was used, which was trained against the 99% SILVA 138 release database^56^, curated with RESCRIPt^57^, trimmed to the V4 region (515F-806R) and weighted^58^ to improve representation of plant-surface communities. Fungal ASVs were classified using the UNITE database (version 9.0, released 2023-07-18)^59^. Contaminant ASVs were identified and removed using decontam, based on DNA extraction negative controls^60^. Additionally, non-target ASVs were filtered: specifically, fruiting body-forming mushrooms were removed from the ITS dataset, and host DNA sequences (mitochondria, chloroplasts) were removed from the 16S dataset. All ASVs that could not be classified to at least the phylum level were also excluded. For downstream diversity analyses, samples were rarefied separately by sample type and amplicon, with rarefaction depths specified in Table 4. Non-phylogenetic alpha (Pielou evenness^61^, observed features, Shannon entropy^62^) and beta diversity metrics (Bray Curtis^63^, Jaccard^64^) were calculated using the q2-diversity plugin. Additionally, k-mer based diversity metrics were calculated^65^. For 16S rRNA gene data, a phylogenetic tree was constructed using fasttree^66^ and MAFFT alignment^67^ with the q2-phylogeny plugin, and this tree was used to calculate phylogeny-informed diversity metrics (weighted UniFrac, unweighted UniFrac^68^) with q2-diversity.

**Table 4:** Rarefaction depths for various sample types and amplicons, and the thereby retained features and samples

Sample Type	Depth	Retained
**ITS**		
Bark	15,000	450,000 (45.13%) features in 30 (90.91%) samples
Soil	5000	220,000 (30.45%) features in 44 (80.00%) samples
Leaves	8000	224,000 (33.53%) features in 28 (90.32%) samples
Berries	10,000	3,400,000 (26.33%) features in 340 (95.51%) samples
Microvinification	4500	423,000 (22.68%) features in 94 (95.92%) samples
**16S**		
Bark	300	7200 (0.72%) features in 24 (72.73%) samples
Soil	1500	70,500 (2.20%) features in 47 (85.45%) samples

Statistical differences in alpha diversity between groups were assessed using Kruskal–Wallis tests via q2-diversity^69^. Permutational multivariate analysis of variance (PERMANOVA) was conducted using the ADONIS function^70,71^ via q2-diversity using multiple distance metrics to ensure robustness across dissimilarity measures. To verify the assumption of homogeneity of group dispersions, we conducted Permutational Analysis of Multivariate Dispersions (PERMDISP)^70^ also via q2-diversity. In cases where PERMDISP indicated significant within-group variance, we complemented the analysis with PCoA plots and additional statistical tests, such as the Mantel test, to further evaluate the structure and separation of groups in multivariate space. Mantel tests were performed using the vegan package^71^, based on Spearman monotonic correlation with 1000 permutations. Importantly, these tests rely on the assumption of independence, yet our spatiotemporal study design may introduce internal autocorrelation and dependencies among samples. We therefore interpreted results with caution and used complementary analysis including visual and multivariate approaches to better reveal the actual underlying structure.

For the prediction of the vineyard from fungal communities of berries we used a supervised random forest machine-learning classifier of the q2-sample-classifier plugin^72^. The models were trained with 10,000 trees. For training the classifier on one year to predict another, we excluded plots that were not sampled across all three years (Plot 11, Plot 18). For the nested cross-validation assessing solely 2021, we excluded plots with fewer than 10 samples.

To predict climate variables from fungal berry microbiomes, we used the machine-learning framework RITME (v1.0.4)^16^. We tested multiple transformation approaches to account for compositional data characteristics—including no transformation, presence/absence encoding, and log-ratio transformations such as Isometric Log-Ratio (ILR), Centered Log-Ratio (CLR), and Additive Log-Ratio (ALR)—as well as exploring different levels of taxonomic aggregation, ranging from ASVs to genus, family, order, and class. Additionally, RITME applies various filtering strategies based on variance and abundance, including thresholding, quantile-based selection, and top-ranked feature selection. We evaluated four distinct supervised machine learning models and types—linear regression, extreme gradient boosting, neural network regression, and random forest regression—using an extensive hyperparameter search for model optimization, and conducting 900 trials per model type for all berry harvest samples.

For microbiome analysis involving taxonomic assignment, we clustered ASVs against the respective reference database at 90% similarity. Performed Multifactor analysis (MFA) to integrate multiple datasets measured on the same set of samples using prince^73^. Prior to integration, each dataset was preprocessed individually to meet the assumptions of linearity and comparability, including appropriate transformations and normalization. The prince MFA implementation performs a principal component analysis (PCA) on each block and automatically scales them, ensuring that all datasets contribute equally to the model structure.

Complementary, we performed Partial least squares discriminant analysis (PLS-DA) using the DIABLO function from mixOmics^74^. The DIABLO model showed high classification accuracy for LC-MS metabolomics data (positive ionization: 94.7%, negative ionization: 92.1%) and moderate accuracy for GC-MS (85.8%) and fungi (76.8%). Bacteria, however, showed lower classification accuracy (59.9%), indicating a weaker contribution of bacterial communities to the model. This is also reflected in the proportion of explained variance, where metabolomics and fungi contributed most to the model variance (Fungi: 52.4%, positive LC-MS 56.0%, negative LC-MC 40.7%, GC-MS: 46.7%), while bacteria accounted for the lowest variance (31.6%).

To interpret overall taxonomic patterns we used HeatTrees from Metacoder^75^. Additionally, to rigorously test differential abundance, we used ANCOM-BC2^76^. All visualizations were generated using matplotlib^77^ and seaborn^78^. Geographic maps were created using geopandas^79^.

### Microvinifications

Microvinifications were performed with five kilograms of Chasselas grapes (*vitis vinifera cv. Chasselas*) from every plot, which were randomly collected a few days prior to commercial harvest. Grapes were kept at 4 °C overnight. The next day, without destemming the fruit, the grapes were pressed twice for 1 min. using a stainless steel BagMixer® 400 (Interscience, St. Nom, France), and the pomace was removed. A first series of samples was taken from the musts of each batch for analysis. At this point, no enological treatment was applied, and sulfur dioxide was not added to the musts to preserve the original microbiome from grapes to musts. The musts were then again kept at 4 °C overnight for sedimentation before removing part of the sediments by a rough racking. Each must sample was filled into two magnum-sized bottles and immediately inoculated with the commercial *Saccharomyces cerevisiae* C19 yeast strain (Levuline C19, Oenofrance, France) which is commonly used in white wines to foster expression of terroir, to start the alcoholic fermentation. Fermentation temperatures were maintained at 18 °C. Must densities were monitored daily. After 14–18 days, as soon as alcoholic fermentations in the individual batches were completed, samples were collected for analysis and the fermented musts of each plot filled into three standard-sized bottles and kept at 18 °C room temperature to naturally induce malolactic fermentations. After several weeks, at the end of malolactic fermentations, a third series of samples was taken from each batch. A final sedimentation was carried out and sulfur dioxide (30 mg/L) was added before bottling the young wines. After a short storage period, a sensory evaluation panel evaluated and scored the wines.

### Sensory analysis

The sensory methodology employed was a descriptive test using a sensory profiling approach known as Quantitative Descriptive Analysis (QDA). The sensory profiling method allows quantification of the perceived intensity of several sensory attributes for each wine. A predefined list of sensory attributes typical for Chasselas from AOC Lavaux was established beforehand (Olfactory intensity, Citrus, Exotic fruit, White fruit, Floral, Green notes, Honey, Milk, Mineral, Reduction, Oxidation, Olfactory complexity, CO_2_, Acidity, Bitterness, Sweetness, Volume in the mouth, Balance, Alcohol, Freshness on the palate, Aromatic length). The wines were served in anonymous black INAO glasses (40 ml) identified by a three-digit code. They were served at a temperature of 12 °C ± 1°C. A comparative profiling method was applied, where tasters had the opportunity to compare the wines against each other. They were required to evaluate the intensity of each sensory attribute on a 10 cm linear scale ranging from “absent” on the left to ‘very intense’ on the right. Tasters marked the perceived intensity level for each wine. Scores were then transformed into ratings ranging from 0 (left endpoint) to 10 (right endpoint). The taster panel consisted of 15 trained panelists. Each taster evaluated the wines in sensory analysis booths compliant with current AFNOR standards. The results were analyzed using a 2-factor Analysis of Variance (ANOVA) (wine factor and judge factor) to identify differences between the wines for each sensory attribute.

### Untargeted metabolomics with HS-SPME-GC-MS

We used untargeted headspace solid-phase microextraction gas chromatography–mass spectrometry (HS-SPME-GC-MS) to analyze volatile organic compounds (VOCs) in grape must and microvinification samples. The headspace analyses were conducted on a Trace 1310 GC (Thermo Scientific) gas chromatograph (GC) coupled to a TSQ 8000 EVO (Thermo Scientific) mass spectrometer (MS) equipped with an RSH (Thermo Scientific) autosampler. Each GC glass vial contained 1 mL of sample with 150 µL buffer (0.5 M Na_2_HPO_4_*2H_2_O, 0.1 M KH_2_PO_4_) and 0.5 g NaCl (>99.5% purity, Sigma Aldrich, Cat No. 71380) and was placed into the RSH tray. Each sample was measured in triplicates as well as additional Air, Buffer and Internal Standard (of each sample type spiked with 10 µL D-Hexanal stock (50 µL/L, >98% purity, Merck, Cat No. 732338-250MG)) as controls (209 vials total). Each vial was incubated at 60 °C for 5 min, while the agitator was set to on for 5 s intervals. The headspace extraction of the sample was subsequently carried out with a Smart SPME Fiber (PAL System Smart SPME Fiber DVB/C-WR/PDMS (Divinylbenzene/Carbon Wide Range/Polydimethylsiloxane), phase 80 µm, fiber thickness 50/30 µm, fiber length 10 mm, color code dark gray) at 40 °C for 15 min. The needle depth in the vial was set to 25 mm. The adsorbed sample was then injected in the SSL inlet set at 240 °C, with a desorption time of 5 min and an injection depth of 40 mm. To avoid carrying over, the SPME-Arrow fiber was pre-conditioned in a dedicated conditioning station (PTV—Back Inlet) for 1 min at 240 °C. GC separation was carried out on a fused silica column (Agilent, DB-WAX GC Column, length 30 m, film thickness 0.25 mm, inner diameter 0.25 µm, format 7 inch cage, Cat No. 122-7032). Helium was the carrier gas used with a constant flow rate of 1.5 mL/min. The inlet temperature was set at 240 °C and with a split ratio of 6.67 (split flow 10 mL/min, spitless time 5 min). A ramped oven program was used, with an initial temperature of 40 °C holding for 3 min, followed by a ramp of 6 °C/min to reach 170 °C. A second ramp was set with 11 °C/min to reach a final temperature of 240 °C with a hold time of 3 min (to completely clean the column). The total run time was 35.1 min and the total analysis time was set to 43 min.

Generated GC-MS data were analyzed in Chromeleon™ Chromatography Data System (CDS) Software (version 7.2). We used the Cobra peak detection algorithm (standard parameters) to integrate peaks and first compared all retrieved peaks (match factor 850) to the Wiley Registry of Mass Spectral Data (ISBN 978-1-119-73632-5) containing over 873,000 reference GC-MS spectra as well as the FFNSC3 (Flavors and Fragrances of Natural and Synthetic Compounds, ISBN: 978-1-119-06964-5) reference library of over 3000 mass spectra. Noise filtering was set to a relative threshold of 5% and we removed all compounds before retention time (RT) 7–8 min as the eluting ethanol is skewing the spectra in this area as well as compounds with a RT > 32 min as these are mostly column contaminations. The confirming peaks (fragments) were manually adjusted according to the assigned compound to update the ion ratio and get more accurate total ion current (TIC) peaks. After an initial automated annotation based on retention times and fragmentation, MS reference spectra were added to each peak and peak annotation performed on a quality MS match within a retention window to provide more security against false negative annotations. Ultimately the generated TIC counts were exported, and the median per sample calculated and normalized using Total Sum Normalization^80^. The Wiley^81^, NIST^82^, and FFNSC^83^ libraries were used to identify volatile compounds, yielding a total of 151 uniquely annotated volatiles.

### Untargeted metabolomics with LC-MS

Untargeted metabolomic analysis of microvinification samples was performed using high-resolution liquid chromatography-mass spectrometry (LC-MS) in both positive and negative ionization modes. LC-MS analyses were conducted using an Orbitrap mass analyzer operating in data-dependent acquisition (DDA) mode, with a mass range of 50–1500 m/z and mass resolutions of 60,000 at 200 m/z for MS1 and 30,000 at 200 m/z for MS2 (see Table 5). Both positive and negative ionization modes were utilized, with stepped collision energies of 10, 30, and 45 NCE. Chromatographic separation was performed using a C18 column (e.g., Acquity BEH C18, 2.1 × 50 mm, 1.7 µm) at a flow rate of 300 µL/min and a temperature of 40 °C. The mobile phases consisted of 0.1% formic acid in water (A) and 0.1% formic acid in acetonitrile (B), with gradient and injection volume parameters specified based on the experimental setup.

**Table 5:** Total number of features identified by each mode of ionization, as well as number of MS1/M2 annotated features and MS2 annotated features

Mode	Features	Annotated Features: MS1/MS2	Annotated Features: MS2
Negative	5355	1389	170
Positive	6205	1942	191

Raw LC-MS data were processed with retention time alignment using ChromAlign, followed by unknown-compound detection with a mass tolerance of 5 ppm, a minimum peak intensity threshold of 10,000, and a minimum of six scans per peak. Peak detection parameters included a chromatographic signal-to-noise threshold of 1.5, a peak width maximum of 0.1 min, and a gap ratio threshold of 0.35. Compound grouping was performed with a 5 ppm mass tolerance and a 0.05-min retention time tolerance, using peak quality assessment that weighted area (3), coefficient of variation (10), and peak shape parameters including jaggedness, modality, and zig-zag index (5 each). Chemical background compounds were identified and hidden based on a comparison of sample-to-blank ratios. For normalization, areas were adjusted using constant median normalization, with blanks excluded from the calculations.

Compound annotation was performed with multiple databases, including mzCloud^84^, mzVault^85^, ChemSpider^86^ with strict mass tolerance thresholds of 5–10 ppm for precursor and fragment ions. Spectral distance and match factor scoring were utilized to assess annotation confidence. Fragment ion annotations were refined through recalibrated libraries and ranked by similarity scores. Database searches for compound identification included BioCyc^87^, the Human Metabolome Database^88^, KEGG^89^, Phenol-Explorer^90^, PlantCyc^91^, Arita Lab 6549 flavonoid structure database^92^, and Natural Products Atlas masslist^93^. ChemSpider searches were performed using predicted compositions, with each compound limited to a maximum of three predicted compositions. Mass tolerance for ChemSpider searches was set to 5 ppm. mzCloud similarity searches utilized cosine matching algorithms to compare ddMS2 spectra, with similarity thresholds set to 50%. Mass range and resolution parameters were adjusted dynamically for accurate spectral interpretation, and isotopic patterns were integrated for precursor evaluation. In addition to compound detection, the analysis included gap-filling across all samples and compound ranking using the mzLogic algorithm^94^. This process utilized chemical background removal and normalization based on the constant median method, excluding blank samples from the calculations. The analysis was further supported by mapping compounds to biological pathways using the Metabolika module.

For subsequent multi-omics analysis, we used the MS2 identified features, and normalized tables with log transformation and Z-score normalization. The annotation accuracy of metabolites showing strong correlations (|corr | >0.9) was further assessed to determine whether a metabolite could be an isomer, based on full matches to the predicted composition, mzCloud search, mzVault search, ChemSpider search, and the mass list search. Potential contamination was also evaluated by examining the sample-to-blank ratio (see Table 6).

**Table 6:** LC-MS identified metabolites that either show strong correlations or rank among the top loadings in the variable plot

Name	Annot. Source: Predicted Compositions	Annot. Source: mzCloud Search	Annot. Source: mzVault Search	Annot. Source: ChemSpider Search	Annot. Source: MassList Search	mzCloud Best Match	mzCloud Best Match Confidence	Ratio: Sample/Blank
5-Hydroxytryptophol	Full match	No results	Full match	Partial match	Partial match	NaN	NaN	1.8
Azelaic acid	Full match	Full match	Full match	Full match	Full match	99.1	10.0	0.8
Norepinephrine	Full match	Full match	Full match	Not the top hit	Not the top hit	88.7	55.2	4.6
N-Methyldioctylamine	Full match	Full match	No results	No results	No results	79.6	49.8	0.4
4-Acetamidobutanoic acid	Full match	Full match	No results	Full match	Not the top hit	78.8	8.9	2.8
N-(5-acetamidopentyl)acetamide	Full match	Full match	No results	Partial match	No results	72.0	50.8	0.4
Hexadecanedioic acid	Full match	Full match	No results	Full match	Full match	71.2	8.6	2.0
C16-0(Palmitoyl)ceramide	Full match	No results	Full match	No results	Partial match	NaN	NaN	1.7
Phenylalanyltyrosine	Full match	No results	Full match	Not the top hit	Partial match	NaN	NaN	2.3
Hydroxyprolyl-Proline	Full match	No results	Full match	Partial match	Partial match	NaN	NaN	4.8
Pro-Phe	Not the top hit	No results	Full match	Partial match	Partial match	NaN	NaN	1.7
1-Palmitoyl-sn-glycero-3-phosphocholine	Not the top hit	No results	Full match	Partial match	Full match	NaN	NaN	0.3
L-Glutamic acid	Full match	Full match	Full match	Full match	Not the top hit	86.4	70.6	0.3
Decaethyleneglycol	Not the top hit	Full match	No results	No results	No results	77.3	73.2	1.0
Undecaethylene glycol	Not the top hit	Full match	No results	No results	No match	75.4	71.8	1.0
Palmitoyl ethanolamide	Full match	Full match	No results	Full match	Full match	88.3	9.4	0.3
2-Amino-1,3,4-octadecanetriol	Full match	Full match	Full match	Full match	Full match	79.8	9.0	1.4
γ-L-glutaminyl-3,4-benzoquinone	Full match	Full match	No results	Partial match	Not the top hit	84.8	9.2	0.9

Metabolites were curated based on identification by at least two annotation sources, a high mzCloud match score (>70%), and a low likelihood of contamination (sample-to-blank ratio < 5).

## Supplementary information

Supplementary Information

## Data Availability

Amplicon sequencing data is available from the Sequence Read Archive (SRA) under accession number PRJEB89111 (16S) and PRJEB89112 (ITS).

## Code availability

All code used in this study is available on GitHub (10.5281/zenodo.17530573).
